# Genetics and Molecular Mechanisms in Oral Squamous Cell Carcinoma: A Narrative Review

**DOI:** 10.3390/medicina62071247

**Published:** 2026-06-28

**Authors:** Cǎtǎlina Ionescu, Ecaterina Tomaziu-Todosia Anton, Viorica Rarinca, Malina Visternicu, Alin Ciobîcă, Bogdan Novac, Daniela Tomița, Mihai Hogas

**Affiliations:** 1 Doctoral School of Biology, Faculty of Biology, “Alexandru Ioan Cuza” University of Iași, 700505 Iași, Romania; catalinaionescu81@yahoo.com (C.I.); rarinca_viorica@yahoo.com (V.R.); malina.visternicu@yahoo.ro (M.V.); 2 “Ioan Haulica” Institute, Apollonia University, 700511 Iasi, Romania; alin.ciobica@uaic.ro; 3 Grigore T. Popa University of Medicine and Pharmacy, University Street, No. 16, 700115 Iasi, Romania; mihaimmh@gmail.com; 4 Department of Obstetrics and Gynecology, Clinical Hospital of Obstetrics and Gynecology Cuza Voda, Cuza Voda Street, No. 34, 700038 Iasi, Romania; 5 Doctoral School of Geosciences, Faculty of Geography and Geology, “Alexandru Ioan Cuza” University of Iași, Carol I Avenue, No 20A, 700505 Iasi, Romania; 6 Department of Biology, Faculty of Biology, “Alexandru Ioan Cuza” University of Iași, 700505 Iasi, Romania; 7 “Olga Necrasov” Center, Department of Biomedical Research, Romanian Academy, 700506 Iasi, Romania; 8 Academy of Romanian Scientists, 050566 Bucharest, Romania; 9 Clinical Department, Apollonia University, Pãcurari Street 11, 700511 Iasi, Romania; daniela.tomita@yahoo.com

**Keywords:** genetics, OSCC, molecular mechanisms, oral squamous-cell carcinoma

## Abstract

Oral squamous-cell carcinoma (OSCC) is the most common form of oral cancer, accounting for over 90% of malignancies in the oral cavity. Its pathogenesis is driven by a complex interplay of genetic alterations, transcriptomic dysregulation, epigenetic modifications, environmental exposures, and tumor microenvironment dynamics. Despite advances in therapy, OSCC remains associated with poor survival due to late diagnosis, therapeutic resistance, and tumor heterogeneity. This narrative review explores genetic determinants and molecular mechanisms underlying OSCC, focusing on recurrent mutations, deregulated pathways, epigenetic control, gene expression changes, insights from cell models, and potential biomarkers for diagnosis and therapy. We integrate findings from the recent literature to provide a comprehensive overview of the current state of research and emerging trends in OSCC genetics.

## 1. Introduction

Oral squamous-cell carcinoma (OSCC) is a highly aggressive malignancy originating from the squamous epithelial cells lining the oral cavity, including the tongue, floor of the mouth, buccal mucosa, gingiva, and lips [1,2]. OSCC accounts for over 90% of oral malignancies, representing a significant public health challenge worldwide due to its high morbidity and mortality rates [3]. Despite advances in surgical techniques, radiotherapy, and chemotherapy, the five-year survival rate for OSCC remains approximately 50–60%, primarily because of late-stage diagnosis, local recurrence, regional metastasis, and the intrinsic heterogeneity of tumors [4].

The etiology of OSCC is multifactorial, arising from complex interactions between environmental exposures, viral infections, and intrinsic genetic susceptibility [5]. Major risk factors include tobacco use, which is strongly associated with DNA adduct formation and mutagenesis; excessive alcohol consumption, which acts synergistically with tobacco to enhance carcinogenesis; betel quid chewing, prevalent in Southeast Asia, contributing to chronic irritation and genotoxicity; and human papillomavirus (HPV) infection, particularly HPV16, which is linked to a subset of OSCCs with distinct molecular profiles.

HPV-positive tumors are characterized by viral oncoprotein-mediated inactivation of p53 and retinoblastoma (Rb) pathways, leading to functional disruption of cell cycle control, along with frequent p16 overexpression, which serves as a surrogate marker of oncogenic HPV activity rather than a direct tumor suppressor effect [6]. In contrast, HPV-negative tumors are predominantly associated with tobacco and alcohol exposure and commonly harbor mutations in *TP53*, *CDKN2A*, and other genes involved in genomic stability and cell cycle regulation [7,8]. Importantly, HPV-driven carcinogenesis is well established in oropharyngeal squamous-cell carcinoma, where it defines a distinct molecular and clinical subtype; however, its contribution to oral cavity OSCC remains controversial, with reported prevalence varying substantially across studies because of differences in anatomical site classification, detection methods, and geographic populations [9]. From a molecular standpoint, HPV-positive tumors tend to display a less complex mutational landscape and a biology driven primarily by viral oncogene expression, whereas HPV-negative OSCC reflects cumulative carcinogen-induced genetic damage [10,11]. Other contributing factors include chronic oral inflammation, poor oral hygiene, and dietary deficiencies [12,13,14,15].

The development of OSCC is a multistep process characterized by the gradual accumulation of genetic and epigenetic alterations. Normal oral epithelium progresses through a series of histopathological stages, from hyperplasia to dysplasia, carcinoma in situ, and finally invasive carcinoma [16]. During this progression, key cellular processes such as cell cycle regulation, apoptosis, DNA repair, and differentiation are progressively disrupted by mutations, chromosomal aberrations, and epigenetic modifications [17]. These molecular changes promote uncontrolled proliferation, invasion of adjacent tissues, angiogenesis, and, eventually, metastasis to regional lymph nodes [18].

A hallmark feature of OSCC is its molecular heterogeneity. Tumors from different patients, or even different regions of the same tumor, often display distinct mutational signatures, gene expression profiles, and epigenetic patterns [19]. This heterogeneity complicates clinical management and contributes to variable responses to standard therapies. For example, mutations in *TP53*, the most frequently altered gene in OSCC, are associated with resistance to chemotherapy and radiotherapy, while alterations in *NOTCH1* and PIK3CA affect differentiation and proliferative capacity. Similarly, epigenetic silencing of tumor suppressor genes via promoter hypermethylation or dysregulated microRNA expression contributes to tumor progression and therapy resistance [20,21].

OSCC is also notable for its interactions with the tumor microenvironment (TME). Chronic inflammation, oxidative stress, and interactions with immune cells and stromal fibroblasts shape tumor evolution, support immune evasion, and influence metastatic potential [22]. Carcinogen-induced oxidative stress leads to DNA damage, while inflammatory cytokines activate survival and proliferative pathways. Additionally, the oral microbiome has emerged as a potential contributor to OSCC pathogenesis, with certain bacterial species promoting inflammation, genotoxicity, and disruption of local immune responses [23,24,25].

Chronic inflammation, oxidative stress, and bidirectional interactions between tumor cells and stromal components collectively shape tumor evolution, promote immune evasion, and enhance metastatic potential [22]. Carcinogen-induced oxidative stress contributes to genomic instability and DNA damage, while inflammatory cytokines such as IL-6 and TNF-α activate pro-survival and proliferative signaling pathways that sustain malignant progression [26].

Within the TME, cancer-associated fibroblasts (CAFs) represent a key stromal population that actively supports OSCC progression. CAFs contribute to extracellular matrix remodeling, secrete growth factors and cytokines, and facilitate tumor invasion through enhanced epithelial-to-mesenchymal transition (EMT). Moreover, CAFs have been implicated in immune modulation by promoting an immunosuppressive milieu, thereby reducing anti-tumor immune surveillance and contributing to resistance to therapy [27,28].

Understanding the genetic and molecular underpinnings of OSCC is essential for improving diagnosis, prognostication, and therapy. Identification of recurrent genetic alterations, dysregulated signaling pathways, and epigenetic modifications provides opportunities for biomarker development, targeted therapies, and personalized medicine approaches [29]. Recent advances in high-throughput sequencing, transcriptomic profiling, and functional studies using OSCC cell lines have considerably expanded our knowledge of the disease, revealing potential molecular targets and therapeutic strategies [21,29].

Several recent reviews have comprehensively summarized the molecular biology of OSCC, including genomic alterations, epigenetic regulation, signaling pathways, and therapeutic strategies [30,31,32]. Rather than reiterating these individual topics, the present review aims to integrate recent evidence across multiple molecular layers—including genetic alterations, transcriptomic dysregulation, epigenetic mechanisms, signaling pathways, tumor microenvironment interactions, microbiome-associated mechanisms, and emerging biomarkers—within a unified OSCC-specific framework. Particular emphasis is placed on the biological crosstalk among these mechanisms and on their translational relevance for biomarker discovery and precision oncology. Furthermore, this review incorporates literature published up to February 2026, providing an updated synthesis of evidence that has emerged after previous comprehensive reviews. [21,29,30]. By bringing together these rapidly evolving areas within a single framework, this review aims to provide a comprehensive and clinically relevant resource for researchers and clinicians involved in OSCC research and management.

**Figure 1:**
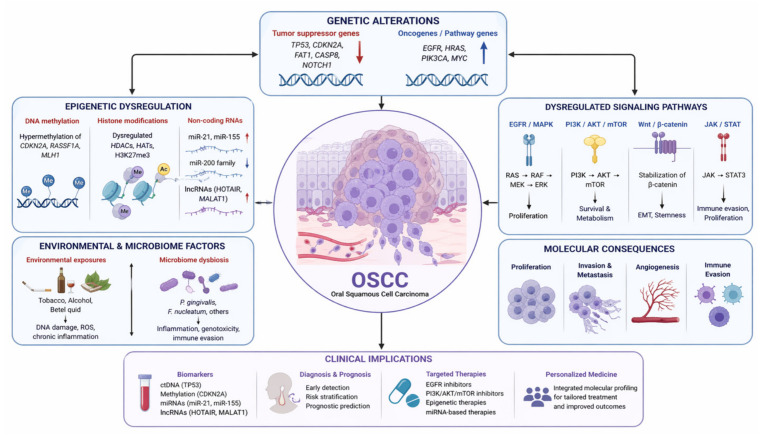
Overview of molecular mechanisms underlying oral squamous-cell carcinoma (OSCC) (partially created with BioRender.com). ↑ = upregulation; ↓ = downregulation.

Figure 1 illustrates the multifactorial molecular landscape of OSCC and its clinical implications. At the center of the figure, OSCC development is depicted as the result of the integration of multiple biological processes. At the genomic level, frequent genetic alterations involve tumor suppressor genes such as *TP53*, *CDKN2A*, *FAT1*, *CASP8*, and *NOTCH1*, as well as oncogenes and pathway-related genes including Epidermal Growth Factor Receptor (EGFR), *HRAS, PIK3CA*, and *MYC*.

Surrounding these genetic events, the figure highlights key epigenetic dysregulation mechanisms, including DNA promoter hypermethylation such as *CDKN2A*, *RASSF1A, MLH1*, altered histone modifications mediated by dysregulated histone deacetylases (HDACs) and histone acetyltransferases (HATs), and aberrant non-coding RNA expression, particularly oncogenic miRNAs (miR-21, miR-155), tumor-suppressive miRNAs (miR-200 family), and long non-coding RNAs such as HOTAIR and MALAT1.

In parallel, major dysregulated signaling pathways are represented, including EGFR/MAPK signaling (RAS–RAF–MEK–ERK axis), PI3K/AKT/mTOR pathway controlling proliferation and metabolic reprogramming, Wnt/β-catenin signaling associated with epithelial–mesenchymal transition and stemness, and JAK/STAT signaling involved in immune evasion and tumor proliferation.

The figure further integrates environmental and microbiome-related factors, such as tobacco, alcohol, and betel quid exposure, which contribute to DNA damage and chronic inflammation, as well as microbial dysbiosis involving species such as *Porphyromonas gingivalis* and *Fusobacterium nucleatum*, which promote inflammation, genotoxic stress, and immune modulation.

These molecular alterations collectively result in key cancer hallmarks, including increased proliferation, invasion and metastasis, angiogenesis, and immune evasion. Finally, the clinical implications section highlights the translational relevance of these mechanisms, including potential biomarkers such as circulating tumor DNA (ctDNA), methylation markers, miRNAs, lncRNAs), diagnostic and prognostic applications, and therapeutic strategies such as EGFR inhibitors, PI3K/AKT/mTOR inhibitors, epigenetic therapies, and emerging miRNA-based approaches, supporting the development of personalized medicine in OSCC.

Beyond illustrating individual molecular alterations, Figure 1 emphasizes that OSCC develops through the convergence of genetic mutations, epigenetic dysregulation, transcriptomic changes, and environmental influences rather than through isolated molecular events. This integrated perspective explains the marked biological heterogeneity of OSCC and highlights why single-target therapeutic strategies often produce limited clinical benefit. Consequently, simultaneous molecular profiling of multiple pathways may provide a stronger basis for precision medicine and biomarker-guided therapeutic selection.

## 2. Research Strategy

A comprehensive literature search was conducted across PubMed, Scopus, and Web of Science databases to identify relevant peer-reviewed studies published up to February 2026. The search strategy utilized combinations of the following Medical Subject Headings (MeSH) terms and keywords: “oral squamous cell carcinoma”, “OSCC”, “multi-omics”, “genomics”, “transcriptomics”, “epigenomics”, “proteomics”, “tumor microenvironment”, and “biomarkers”.

Studies were included if they provided mechanistic insights into the molecular landscape of OSCC, clinical correlations, or multi-omics integration. Exclusion criteria consisted of: (1) studies focusing exclusively on non-oral HNSCC subsites (aryngeal, hypopharyngeal, or HPV-positive oropharyngeal carcinomas) without distinct OSCC cohorts; (2) duplicate publications; (3) case reports, editorials, or conference abstracts; and (4) studies with unverified cell lines or lack of ethical approval. A total of 186 unique articles were ultimately selected for qualitative synthesis based on these criteria.

## 3. Molecular Mechanisms of OSCC

The molecular heterogeneity of OSCC is reflected in a diverse spectrum of genomic alterations involving somatic mutations, chromosomal abnormalities, copy number variations, and, less frequently, germline variants. Rather than acting independently, these alterations cooperate to disrupt key regulatory networks controlling cell proliferation, apoptosis, differentiation, and genomic stability, thereby driving tumor initiation and progression [33]. These genetic alterations drive tumor initiation, progression, and heterogeneity, affecting cellular processes such as proliferation, apoptosis, differentiation, DNA repair, and signal transduction [34]. In OSCC, no single mutation is solely responsible for carcinogenesis; instead, a cooperative accumulation of genetic changes in oncogenes, tumor suppressor genes, and regulatory pathways underlies malignant transformation. High-throughput sequencing studies and multi-center genomic analyses have provided comprehensive maps of recurrent genetic alterations in OSCC, revealing patterns of mutational hotspots, pathway-specific dysregulation, and correlations with clinical outcomes [30,35,36].

### 3.1. Genetic Alterations in OSCC

#### 3.1.1. *TP53*

Mutations in *TP53*, encoding the tumor suppressor p53, are the most frequent genetic events in OSCC, occurring in approximately 50–70% of oral cavity OSCC cases [29]; however, reported frequencies vary considerably across cohorts, ranging from ~30–50% in certain Asian OSCC populations to ~70–85% in HPV-negative Head and Neck Squamous Cell Carcinoma (HNSCC) datasets, including The Cancer Genome Atlas (TCGA) analyses [37,38]. *TP53* is commonly referred to as the “guardian of the genome” due to its critical role in maintaining genomic integrity through regulation of cell cycle checkpoints, apoptosis, DNA repair, and senescence [21,39]. Mutations frequently target the DNA-binding domain, resulting in loss of transcriptional activity and dominant-negative effects. Among these, recurrent hotspot mutations such as R175H, R248Q, and R273H are among the most frequently reported alterations in OSCC and HNSCC and represent key drivers of p53 dysfunction [40]. Dysfunctional p53 permits the survival of genetically damaged cells, leading to genomic instability, clonal evolution, and increased aggressiveness. Clinically, *TP53* mutations are associated with resistance to chemotherapeutic agents and radiotherapy, as well as poorer prognosis. As extensively documented across various malignancies, including breast cancer models [41,42,43] and increasingly verified in head and neck datasets, specific *TP53* mutation types (missense versus nonsense) correlate with distinct tumor behaviors and metastatic potential. While nonsense mutations generally result in a complete loss of p53 expression or function, certain missense mutations can exert dominant-negative effects and acquire gain-of-function (GOF) properties. These canonical hotspot mutants, including R175H, R248Q, and R273H, are particularly well characterized for actively promoting cell proliferation, invasion, metastasis, and therapy resistance, thereby contributing to a highly aggressive tumor phenotype [40,44].

It is critical to distinguish between general HNSCC cohorts and strict OSCC datasets regarding *TP53* mutations. While *TP53* alterations are ubiquitous across HNSCC, their prevalence and mutational spectrum in OSCC are heavily influenced by geographic risk factors. In Western cohorts (primarily tobacco and alcohol-driven), *TP53* mutations are often associated with canonical disruptive transitions. Conversely, in Asian cohorts where betel quid chewing is predominant, *TP53* mutations frequently co-occur with distinct transversion patterns and are closely tied to a low HPV prevalence, highlighting that OSCC represents a distinct etiopathogenetic entity compared to HPV-positive oropharyngeal carcinomas.

#### 3.1.2. *CDKN2A* (p16INK4a)

The *CDKN2A* gene encodes the p16INK4a protein, a crucial regulator of the G1/S cell cycle checkpoint [45]. Genomic studies indicate that *CDKN2A* alterations occur in approximately 10–20% of OSCC cases, although additional functional inactivation through promoter hypermethylation substantially increases the overall frequency of p16 pathway disruption [46]. Inactivation of *CDKN2A* occurs via point mutations, homozygous deletions, or promoter methylation, disrupting the CDK4/6-Rb pathway and enabling unchecked cell cycle progression. Loss of p16 function is observed in a significant subset of OSCC cases, particularly those linked to tobacco and alcohol exposure. Clinically, *CDKN2A* inactivation is associated with early tumor development and may serve as a biomarker for pre-malignant lesions and risk stratification [45,47,48].

#### 3.1.3. RAS Family (*HRAS*, *KRAS*, *NRAS*)

Mutations in RAS proto-oncogenes, most frequently *HRAS*, are less common in OSCC than in other cancers but remain critical in driving oncogenic signaling. *HRAS* mutations typically occur at codons 12, 13, or 61, resulting in constitutively active RAS proteins that promote uncontrolled proliferation through the MAPK/ERK and *PI3K/AKT* pathways [49,50]. These mutations contribute to enhanced cell survival, motility, and angiogenesis. Although HRAS mutations are less frequent, their presence may cooperate with *TP53* and PIK3CA alterations to accelerate tumor progression [51].

#### 3.1.4. *PIK3CA*

*PIK3CA,* encoding the p110α catalytic subunit of PI3K, is frequently mutated or amplified in OSCC [52]. Activating *PIK3CA* mutations hyperactivate the PI3K/AKT/mTOR signaling axis, promoting cell survival, proliferation, and resistance to apoptosis. *PIK3CA* mutations often coexist with other driver mutations, particularly *TP53*, contributing to aggressive phenotypes and therapy resistance. PI3K pathway inhibitors are currently under investigation as targeted therapies for OSCC [53,54].

#### 3.1.5. *NOTCH1*

Unlike *TP53* and *CDKN2A* alterations, whose pathogenic roles are well established, the role of *NOTCH1* remains incompletely understood and appears highly context-dependent [55]. The role of *NOTCH1* in OSCC is context-dependent, acting as either a tumor suppressor or oncogene depending on cellular and environmental context. Loss-of-function mutations often result in impaired differentiation, while gain-of-function mutations can promote tumorigenesis. Altered *NOTCH1* signaling influences squamous differentiation, stemness, and interaction with other pathways, such as p53 and PI3K. Dysregulated *NOTCH1* is also associated with poor differentiation and higher tumor grade [55,56,57].

Beyond the major driver genes described above, large-scale genomic studies of OSCC and HNSCC have identified several additional recurrently altered genes that contribute to tumor biology and heterogeneity. These include *CTNNB1, NSD1, KMT2D*, and *AJUBA*, which are involved in key regulatory processes such as Wnt/β-catenin signaling, chromatin remodeling, epigenetic regulation, and cell adhesion [38,58,59,60,61,62]. Although less frequently mutated compared to canonical drivers such as *TP53* or *CNKN2A*, these alterations are increasingly recognized for their role in shaping tumor behavior, influencing differentiation states, and contributing to intratumoral heterogeneity and disease progression [38].

### 3.2. Additional Genetic Alterations

In addition to canonical driver genes, OSCC frequently harbors mutations and amplifications in several other genes that contribute to tumor progression. *FAT1*, a tumor suppressor involved in maintaining cell adhesion, is often mutated, facilitating EMT and promoting invasive behavior [63]. *CASP8*, a key regulator of apoptosis, is frequently inactivated, allowing survival of cells with accumulated DNA damage [64]. Amplifications of EGFR enhance proliferative and survival signaling, and overexpression of this receptor is a hallmark feature in a majority of OSCC cases [65]. Similarly, *MYC* amplification drives uncontrolled proliferation and metabolic reprogramming, supporting tumor growth and adaptation [66].

### 3.3. Cooperation of Mutations and Tumor Evolution

The recurrent genetic alterations described above collectively define the molecular profile of OSCC and illustrate the multistep nature of oral carcinogenesis. *TP53* mutations, the most frequent genetic events in OSCC, play a central role in disrupting genomic stability and cellular homeostasis [29,67].

Refs. [68,69] Within this molecular context, *TP53* mutations represent the most frequent genomic alteration and a pivotal early event in oral carcinogenesis, compromising genomic integrity and facilitating apoptosis evasion. Inactivation or deletion of *CDKN2A* further disrupts cell cycle regulation, promoting uncontrolled proliferation [68,69]. Activating mutations in HRAS and PIK3CA sustain constitutive proliferative and survival signaling through the MAPK and PI3K/AKT pathways [70,71]. Alterations affecting *NOTCH1* contribute to impaired epithelial differentiation and tumor heterogeneity [72], whereas loss-of-function mutations in *FAT1* and *CASP8* promote epithelial-to-mesenchymal transition, invasion, and apoptosis resistance [73,74]. Furthermore, amplification or overexpression of EGFR and *MYC* reinforces proliferative signaling and metabolic reprogramming, thereby supporting tumor growth and disease progression [38,75,76]. These genetic events often act cooperatively, highlighting the multigenic nature of OSCC pathogenesis and underscoring the importance of integrated molecular profiling to guide targeted therapeutic strategies. The major recurrent genetic alterations in OSCC and their functional consequences are summarized in Table 1.

## 4. Gene Expression and Transcriptomic Dysregulation

OSCC is characterized by profound alterations in gene expression that contribute to tumor initiation, progression, and heterogeneity. Transcriptomic changes involve not only protein-coding genes but also non-coding RNAs, such as microRNAs (miRNAs) and long non-coding RNAs (lncRNAs), which modulate post-transcriptional gene regulation. These dysregulations affect fundamental cellular processes, including proliferation, apoptosis, differentiation, invasion, and angiogenesis [31,82,83].

### 4.1. Oncogene Upregulation

High-throughput RNA sequencing and microarray analyses of OSCC tissues and cell lines consistently demonstrate the upregulation of key oncogenes involved in cellular proliferation and survival [84]. Notably, the EGFR is overexpressed in the majority of OSCC cases, promoting proliferation, survival, and migration through activation of downstream MAPK, PI3K/AKT, and JAK/STAT signaling pathways. EGFR overexpression is associated with poor prognosis and aggressive tumor phenotypes [29,85,86]. Cyclin D1 (*CCND1*) is similarly upregulated, accelerating the G1/S transition of the cell cycle and contributing to uncontrolled proliferation, with *CCND1* amplification frequently observed in high-grade tumors [87]. The transcription factor *MYC*, when overexpressed, enhances cellular proliferation, metabolic reprogramming, and stemness properties. Additional oncogenes, including *BCL2, MMP9*, and *VEGF*, facilitate anti-apoptotic signaling, extracellular matrix remodeling, and angiogenesis, respectively, representing conserved core mechanisms that support the aggressive behavior of oral tumors [88,89].

### 4.2. Tumor Suppressor Downregulation

In contrast, OSCC is characterized by the downregulation of tumor suppressor genes that normally restrain proliferation and maintain genomic stability. *TP53* target genes, although frequently mutated, show reduced expression of downstream effectors such as p21 (*CDKN1A*), *GADD45*, and *BAX*, leading to impaired DNA damage response and diminished apoptosis [90,91]. *CDKN2A* (p16INK4a) is downregulated not only through genetic inactivation but also via epigenetic silencing and transcriptional repression. Loss of *PTEN* expression activates PI3K/AKT signaling, further enhancing cell survival and proliferation [92,93].

### 4.3. Transcriptomic Subtypes of OSCC

Large-scale transcriptomic profiling has revealed that OSCC is not a single molecular entity but comprises several distinct molecular subtypes, each associated with specific biological behaviors and clinical outcomes. The classical or keratinized subtype is characterized by high expression of epithelial differentiation genes such as *KRT1* and *KRT10* and is often associated with tobacco exposure [31]. The mesenchymal or invasive subtype exhibits elevated EMT markers including *VIM, SNAI2*, and *ZEB1*, along with increased migratory capacity and poor prognosis [94]. An immune-rich subtype displays upregulation of immune-related genes such as *CD8A*, *IFNG*, and *CXCL9* and may be more responsive to immunotherapy [95].

### 4.4. Role of Non-Coding RNAs

Non-coding RNAs play pivotal roles in OSCC by regulating post-transcriptional gene expression and epigenetic programs involved in proliferation, invasion, epithelial–mesenchymal transition, and therapeutic resistance. Although some mechanistic evidence derives from broader HNSCC studies, the findings summarized below are supported by studies performed in OSCC tissues or cell models whenever available. MicroRNAs (miRNAs) are short RNAs that negatively regulate mRNA translation; dysregulated examples include miR-21, which has been shown to suppress *PTEN* and *PDCD4* to enhance proliferation and invasion across multiple tumors including glioblastoma, a mechanism heavily mirrored in oral malignancies, while the miR-200 family controls epithelial–mesenchymal transition [96,97]. Long non-coding RNAs (lncRNAs) such as HOTAIR and MALAT1 influence chromatin remodeling, gene expression, and metastasis, with elevated HOTAIR levels linked to poor prognosis and lymph node metastasis [98,99].

### 4.5. Clinical Implications of Transcriptomic Dysregulation

Gene expression signatures have demonstrated utility as prognostic and predictive biomarkers. High expression of *CCND1* or *EGFR* correlates with advanced tumor stage, lymph node metastasis, and reduced overall survival [100]. EMT-associated gene expression profiles predict invasive behavior and metastatic potential. Furthermore, immune-related expression signatures may identify patients with higher likelihood of responding to immune checkpoint inhibitors [101].

Overall, transcriptomic dysregulation in OSCC represents a complex interplay of oncogene activation, tumor suppressor repression, and non-coding RNA-mediated regulation. These molecular alterations not only drive tumorigenesis but also shape the heterogeneity, metastatic potential, and therapeutic response of OSCC [102].

Of clinical relevance, transcriptomic subtypes of OSCC may also have implications for therapeutic stratification, particularly in the context of immune checkpoint inhibition. Immune-inflamed gene expression profiles characterized by increased *CD8A*, *IFNG*, and *CXCL9* expression have been associated with a higher likelihood of response to PD-1/PD-L1 blockade, suggesting a potential link between transcriptomic heterogeneity and immunotherapy outcomes in HNSCC, including OSCC [38,103,104,105].

## 5. Epigenetic Regulation in OSCC

Epigenetic mechanisms contribute to OSCC pathogenesis by modulating gene expression without altering the underlying DNA sequence [32]. Epigenetic mechanisms include DNA methylation, histone modifications, chromatin remodeling, and non-coding RNAs, all of which contribute to tumor initiation, progression, and heterogeneity [106]. These modifications can lead to silencing of tumor suppressor genes, activation of oncogenes, and dysregulation of signaling pathways, thereby complementing somatic mutations in driving carcinogenesis.

### 5.1. DNA Methylation Patterns

DNA methylation, particularly at CpG islands within gene promoters, represents a central epigenetic mechanism in OSCC. Hypermethylation of tumor suppressor gene promoters results in transcriptional silencing, disrupting critical pathways that regulate the cell cycle, DNA repair, apoptosis, and cellular differentiation [107]. Prominent examples include *CDKN2A* (p16INK4a), whose promoter hypermethylation reduces expression and abrogates G1/S checkpoint control, thereby promoting uncontrolled proliferation [108]. Similarly, *RASSF1A* inactivation through hypermethylation impairs apoptotic signaling, enhancing cell survival, while *APC* methylation dysregulates Wnt signaling, favoring tumor growth and proliferation. Silencing of *MLH1* compromises mismatch repair, leading to increased mutation accumulation and genomic instability [109]. Numerous studies have demonstrated that these hypermethylation patterns correlate with tumor stage, grade, and metastatic potential, highlighting their potential utility as prognostic biomarkers [110,111,112,113].

### 5.2. Histone Modifications and Chromatin Remodeling

Post-translational modifications of histones, including acetylation, methylation, phosphorylation, and ubiquitination, regulate chromatin accessibility and transcriptional activity. Dysregulation of histone-modifying enzymes is frequently observed in OSCC [110,114]. Overexpression of HDACs promotes chromatin condensation and silencing of tumor suppressor genes, whereas loss or dysfunction of HATs reduces acetylation and represses pro-apoptotic and differentiation-associated genes [115]. Aberrant histone methylation patterns, such as H3K27me3, contribute to gene silencing and the maintenance of stem-like tumor phenotypes [116].

### 5.3. MicroRNAs and Non-Coding RNAs

Non-coding RNAs, particularly microRNAs (miRNAs) and long non-coding RNAs (lncRNAs), play pivotal roles in post-transcriptional and epigenetic regulation in HNSCC, including OSCC, although many findings are based on broader HNSCC datasets rather than OSCC-specific data [117]. Also, miR-21, frequently overexpressed, targets tumor suppressors such as *PTEN* and *PDCD4,* promoting proliferation, invasion, and resistance to apoptosis. miR-155 modulates immune signaling and EMT, contributing to metastatic potential, while downregulation of the miR-200 family enhances EMT and tumor invasiveness [118,119]. lncRNAs such as HOTAIR and MALAT1 regulate chromatin states and gene expression, influencing metastasis and stemness. Dysregulated networks of non-coding RNAs can simultaneously modulate multiple oncogenic pathways, amplifying tumor progression.

Some studies have delineated the major epigenetic mechanisms contributing to OSCC pathogenesis. Analysis of HNSCC and OSCC tumor tissues demonstrated that promoter hypermethylation of tumor suppressor genes, including *CDKN2A*, *RASSF1A*, and *MLH1*, leads to transcriptional silencing and correlates with tumor stage [120]. Histone modification studies in OSCC cells and tissues revealed that dysregulated histone deacetylases, particularly HDAC1 and other HDACs, repress pro-apoptotic genes, while PRC2/EZH2-mediated H3K27me3 silences differentiation-associated genes, collectively enhancing tumor aggressiveness [121]. Dysregulation of microRNAs in OSCC tissues compared with normal mucosa showed that upregulation of miR-21 and miR-155 promotes proliferation and invasion, whereas downregulation of the miR-200 family facilitates EMT [122]. Additionally, long non-coding RNAs, such as HOTAIR and MALAT1, are overexpressed in CAL27 cells, with elevated levels associated with enhanced invasion, EMT, metastasis, and poor prognosis [123]. Collectively, these findings highlight the coordinated involvement of DNA methylation, histone modifications, and non-coding RNAs in OSCC, emphasizing their potential as diagnostic, prognostic, and therapeutic targets. Representative studies highlighting key epigenetic mechanisms in OSCC are summarized in Table 2.

## 6. Dysregulated Signaling Pathways

**Figure 2:**
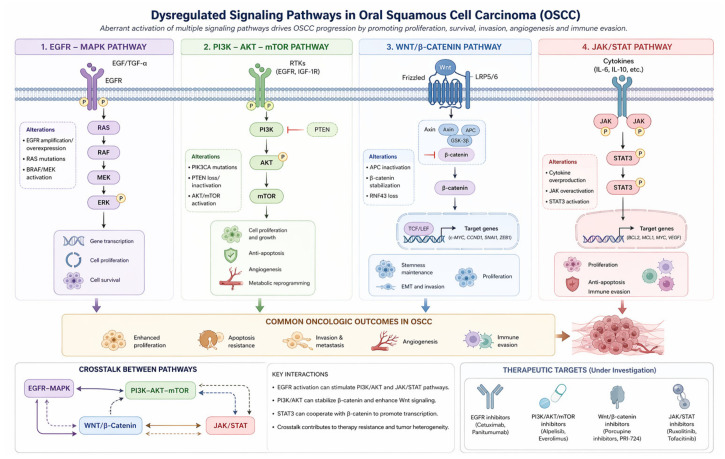
Dysregulated signaling pathways in OSCC (partially created with BioRender.com).

In OSCC, tumor progression is driven by the aberrant activation of multiple interconnected intracellular signaling pathways that control essential processes such as cell proliferation, survival, apoptosis evasion, invasion, metastasis, angiogenesis, and immune evasion. Figure 2 summarizes the main oncogenic axes involved in these processes.

Specifically, the EGFR–MAPK pathway is frequently activated through EGFR overexpression or amplification, leading to activation of the RAS–RAF–MEK–ERK cascade and promoting the transcription of genes involved in cell proliferation and survival. This axis represents one of the main determinants of tumor growth in OSCC.

The PI3K–AKT–mTOR pathway is activated through receptor tyrosine kinase signaling (including EGFR) and by genetic alterations such as PI3K mutations or *PTEN* loss of function. Activation of this cascade supports cell growth, inhibition of apoptosis, angiogenesis, and metabolic reprogramming, significantly contributing to tumor aggressiveness.

The WNT/β-catenin axis regulates processes of differentiation, proliferation, and maintenance of cancer stem cell properties. In OSCC, activation of this pathway through mutations or dysregulation of the β-catenin degradation complex leads to nuclear translocation of β-catenin and activation of target genes involved in EMT and invasion.

The JAK/STAT pathway is stimulated by pro-inflammatory cytokines and contributes to proliferation, cell survival, and immune evasion through the activation of transcription of genes associated with inflammation and anti-apoptotic processes.

The figure also highlights the existence of an extensive crosstalk network among these signaling pathways, particularly between EGFR–MAPK, PI3K–AKT–mTOR, WNT/β-catenin, and JAK/STAT, which amplifies oncogenic signals and contributes to tumor heterogeneity and therapy resistance.

Overall, these dysregulations converge toward common oncogenic phenotypes, including increased proliferation, resistance to apoptosis, invasion and metastasis, angiogenesis, and immune evasion. The figure also includes the main therapeutic targets under investigation, aimed at blocking these signaling cascades [123,124].

Importantly, Figure 2 illustrates that extensive crosstalk among these pathways generates compensatory signaling mechanisms capable of maintaining tumor growth even when one pathway is therapeutically inhibited. This network-based organization provides a biological rationale for combined targeted therapies and supports the development of multi-pathway therapeutic approaches instead of single-agent inhibition.

### 6.1. EGFR-Mediated Signaling

The Epidermal Growth Factor Receptor (EGFR) is overexpressed in approximately 80–90% of OSCC tumors [65]. Ligand binding induces receptor dimerization and autophosphorylation, triggering multiple downstream signaling cascades [124]. The MAPK/ERK pathway promotes transcription of genes controlling proliferation and differentiation, and its constitutive activation drives uncontrolled cell cycle progression [125]. The PI3K/AKT pathway enhances cell survival, inhibits apoptosis, and contributes to therapeutic resistance, while JAK/STAT signaling mediates the expression of inflammatory and immune regulatory genes, influencing both the tumor microenvironment and immune evasion [126,127]. Dysregulation of EGFR is strongly correlated with advanced tumor stage, nodal metastasis, and poor prognosis, establishing EGFR as a central target for monoclonal antibodies and tyrosine kinase inhibitors in clinical trials [128,129].

### 6.2. PI3K/AKT/mTOR Axis

The PI3K/AKT/mTOR pathway is frequently dysregulated in OSCC, often through activating mutations or amplification of *PIK3CA*, which enhances AKT phosphorylation and downstream mTOR activation, or through loss of *PTEN*, a negative regulator of PI3K signaling [54,130]. Aberrant activation of this axis drives increased cell proliferation and metabolism via mTOR-mediated protein synthesis and nutrient sensing, resistance to apoptosis through AKT-mediated inhibition of pro-apoptotic factors such as BAD and caspase-9, and promotion of angiogenesis via upregulation of *VEGF*. Targeted therapies against PI3K, AKT, and mTOR are under investigation, particularly in tumors harboring PIK3CA alterations or *PTEN* loss [130,131,132].

### 6.3. Wnt/β-Catenin Signaling

The Wnt/β-catenin pathway regulates cell fate, differentiation, and stemness across various malignancies. While these fundamental oncogenic mechanisms, including how dysregulation stabilizes β-catenin, facilitating nuclear translocation and transcription of proliferation-associated genes (*MYC*, *CCND1)* and EMT regulators (*SNAI1*, *ZEB1*)—have been well-characterized in general cancer models [133], they play a critical role in OSCC progression as well. Aberrant Wnt activation enhances invasive capacity, metastatic potential, and the maintenance of cancer stem-like cells in oral tumors. Furthermore, within the context of OSCC, Wnt signaling interacts with other oncogenic pathways, including PI3K/AKT and EGFR, establishing positive feedback loops that reinforce tumor progression [134,135,136].

### 6.4. JAK/STAT Pathway

In OSCC, the JAK/STAT pathway integrates cytokine and growth factor signals to regulate immune responses, proliferation, and survival. Persistent activation of STAT3, commonly induced by OSCC-specific upstream drivers such as EGFR or IL-6 signaling, drives the transcription of anti-apoptotic genes (*BCL2*, *MCL1*) as well as proliferative oncogenes including *MYC* and *CCND1* in oral tumor cells. Furthermore, within the OSCC microenvironment, STAT3 signaling modulates tumor-immune crosstalk, promoting immune evasion by suppressing cytotoxic T-cell activity. Consequently, this aberrant JAK/STAT activation is closely associated with OSCC aggressiveness, nodal metastasis, and poor clinical outcomes [137,138,139,140].

### 6.5. Crosstalk Between Pathways

Comprehensive analyses of OSCC signaling networks highlight recurrent dysregulation across multiple oncogenic pathways. The EGFR-MAPK/PI3K/STAT axis is frequently activated through overexpression or ligand-mediated receptor stimulation, driving enhanced proliferation, survival, and immune evasion [141]. Dysregulation of the PI3K/AKT/mTOR pathway, often resulting from *PIK3CA* mutations or *PTEN* loss, promotes resistance to apoptosis, metabolic reprogramming, and angiogenesis, further supporting tumor progression [142]. Aberrant Wnt/β-catenin signaling, characterized by stabilization of β-catenin, facilitates EMT, maintenance of stem-like properties, and increased invasive potential [143]. Persistent activation of the JAK/STAT pathway, particularly STAT3, drives transcription of genes involved in proliferation, anti-apoptotic signaling, and immune modulation, thereby contributing to tumor aggressiveness and immune evasion [144]. Collectively, these findings emphasize the coordinated interplay between signaling pathways in OSCC, underlining the need for multi-targeted therapeutic strategies to effectively counteract compensatory oncogenic signaling. Key dysregulated signaling pathways in OSCC are summarized in Table 3.

## 7. OSCC Cell Line Models and Functional Studies

Cell line models are indispensable tools for investigating the molecular mechanisms underlying OSCC. These models provide a controlled environment to study genetic, epigenetic, and transcriptomic alterations, as well as to assess functional consequences of specific molecular changes [21,143].

### 7.1. Common OSCC Cell Lines

Several established OSCC cell lines are extensively employed in molecular and translational research. SCC15 and SCC25, derived from human tongue squamous-cell carcinomas, retain key features of the primary tumors, including *TP53* mutations and EGFR overexpression, and are commonly used for studies of transcriptomics, proliferation, invasion, and drug sensitivity. CAL27, also originating from tongue carcinoma, exhibits constitutive activation of PI3K/AKT and MAPK signaling, making it suitable for pathway-specific experiments and therapeutic testing. HSC-3, established from a primary tongue squamous-cell carcinoma, is widely used as a model for investigating invasion, migration, and EMT-related mechanisms due to its highly invasive phenotype [145,146,147].

### 7.2. Functional Approaches

Functional studies in OSCC cell lines utilize multiple strategies to manipulate gene expression or modulate signaling pathways. RNA interference (siRNA/shRNA) enables targeted knockdown of specific genes, facilitating analysis of their roles in proliferation, apoptosis, invasion, and migration—for example, silencing *NOTCH1* or EGFR reduces proliferative capacity and EMT [148,149]. CRISPR/Cas9 gene editing allows precise knockout or correction of genetic loci, validating the causal roles of mutations such as *TP53* or *PIK3CA* in tumor behavior [150]. Overexpression assays introduce oncogenes or tumor suppressors to assess gain-of-function effects; for instance, restoring *PTEN* expression in *PIK3CA*-mutant cell lines can reinstate apoptotic pathways and suppress AKT signaling [151].

### 7.3. Applications in Translational Research

Functional studies using OSCC cell lines have provided critical insights into the molecular mechanisms driving tumor progression and therapeutic responses. Transcriptome profiling of SCC15 and SCC25 cells enabled identification of somatic mutations and expression patterns, including alterations in *TP53* and *CCND1*, which mirror patient tumor biology [152]. Investigation of *NOTCH1* in CAL27 and HSC-3 cells demonstrated that inhibition of this pathway reduces proliferation and downregulates EMT markers, highlighting its role in tumor aggressiveness [153]. Modulation of microRNAs in SCC25 cells revealed that miR-21 inhibition increases *PTEN* expression and diminishes invasive potential, confirming its oncogenic function [154]. Targeted inhibition of the *PIK3CA*/AKT pathway in CAL27 cells decreased proliferation and sensitized cells to apoptosis, underscoring the therapeutic potential of pathway-specific interventions [155]. Finally, epigenetic therapies using histone deacetylase (HDAC) inhibitors in OSCC cell lines restored tumor suppressor expression and reduced proliferation, demonstrating the utility of epigenetic modulation as a complementary treatment strategy [156]. Collectively, these studies exemplify how OSCC cell lines can model genetic, epigenetic, and signaling dysregulation, facilitating mechanistic validation and preclinical therapeutic evaluation. Representative functional studies using OSCC cell lines are summarized in Table 4.

## 8. Integration with Environmental and Microbiome Factors

OSCC arises from a complex interplay between genetic predisposition, epigenetic dysregulation, and environmental exposures. Environmental factors not only act as direct carcinogens but also modulate the oral microbiome, chronic inflammation, and oxidative stress, creating a permissive milieu for malignant transformation.

### 8.1. Environmental Exposures

Environmental factors play a pivotal role in the etiology and progression of OSCC. Tobacco smoke, which contains polycyclic aromatic hydrocarbons, nitrosamines, and reactive oxygen species (ROS), induces DNA adduct formation, mutagenesis—particularly in *TP53*—and epigenetic alterations, thereby promoting genomic instability [160]. Chronic exposure further enhances proliferation, angiogenesis, and immune evasion [161,162]. Alcohol consumption, metabolized to the DNA-damaging agent acetaldehyde, acts synergistically with tobacco to increase mutation burden, impair DNA repair, and disrupt gene expression [163]. In South and Southeast Asia, betel quid chewing represents a significant risk factor; the arecoline in betel quid induces ROS, DNA cross-linking, and epigenetic modifications that contribute to OSCC development [14,161,163]. Chronic inflammation, as observed in periodontal disease and oral infections, triggers sustained activation of inflammatory pathways, such as NF-κB, which further promotes proliferation, angiogenesis, and genomic instability [164].

### 8.2. Oral Microbiome and Dysbiosis

The oral microbiome has emerged as a potential contributor to OSCC pathogenesis; however, whether microbial dysbiosis represents a causal factor, a consequence of tumor development, or both remains under active investigation. Several studies support an association between oral microbial dysbiosis and OSCC; however, current evidence does not yet establish a direct causal relationship. Pathogens such as *Porphyromonas gingivalis* and *Fusobacterium nucleatum* induce pro-inflammatory cytokines, including *IL-6* and *TNF-α*, which activate *STAT3*, NF-κB, and MAPK pathways, creating a pro-tumorigenic microenvironment [164,165,166,167]. Certain bacterial toxins generate reactive oxygen and nitrogen species, causing DNA damage and epigenetic alterations in epithelial cells. Dysbiosis also impairs anti-tumor immunity by suppressing cytotoxic T-cell activity, thereby facilitating immune evasion [168]. Moreover, microbial metabolism of tobacco- and alcohol-derived compounds can enhance their mutagenic potential [169,170]. Recent studies have identified specific microbial signatures associated with OSCC progression, metastatic propensity, and therapeutic response, suggesting that microbiome profiling may serve as a biomarker for early detection and risk stratification [171,172].

### 8.3. Gene–Environment–Microbiome Interactions

The pathogenesis of OSCC reflects complex interactions among genetic alterations, epigenetic regulation, and environmental or microbial exposures. Mutations in *TP53* and silencing of *CDKN2A* sensitize epithelial cells to ROS-induced DNA damage, while chronic inflammation and microbiome-derived toxins amplify oxidative stress and drive epigenetic dysregulation, such as hypermethylation of tumor suppressor genes [31]. Aberrant activation of signaling pathways, including EGFR, PI3K/AKT, NF-κB, and STAT3, can be triggered both by intrinsic genetic alterations and by extrinsic environmental or microbial stimuli [173].

## 9. Clinical Implications and Emerging Biomarkers

The integration of genetic, epigenetic, and transcriptomic data in OSCC has significant clinical implications for early detection, prognosis, and personalized therapy. Molecular biomarkers derived from tumor tissue, circulating DNA, or saliva are increasingly explored as tools for diagnosis, risk stratification, and therapeutic guidance.

### 9.1. Diagnostic Biomarkers

Early detection of OSCC remains challenging due to asymptomatic lesions and clinical heterogeneity. Molecular biomarkers offer a complementary approach to conventional histopathology. ctDNA enables minimally invasive detection of specific mutations, such as *TP53* and *PIK3CA*, or aberrant methylation patterns in plasma, providing information on tumor burden and recurrence [174]. Promoter hypermethylation of tumor suppressor genes, including *CDKN2A*, *RASSF1A,* and *MLH1*, in oral epithelial cells or saliva correlates with early carcinogenic changes. Additionally, dysregulated non-coding RNAs, such as miR-21, miR-155, HOTAIR, and MALAT1, can be detected in saliva or plasma and serve as promising non-invasive biomarkers for early OSCC detection [175].

### 9.2. Therapeutic Implications

Although several molecular biomarkers have shown promising results in preclinical and retrospective studies, only a limited number have achieved sufficient validation for routine clinical implementation. Targeted therapies addressing genetic and epigenetic alterations are under active investigation in OSCC. EGFR-directed therapies, including monoclonal antibodies such as cetuximab and small-molecule tyrosine kinase inhibitors, have demonstrated variable clinical efficacy, largely due to tumor heterogeneity and the development of acquired resistance [37]. In clinical settings, cetuximab remains the only EGFR-targeted agent with established benefit in recurrent/metastatic HNSCC. Most clinical evidence originates from HNSCC clinical trials, whereas OSCC-specific prospective studies remain limited [176].

PI3K/AKT/mTOR inhibitors are explored in tumors harboring *PIK3CA* activation or *PTEN* loss [31,177]. However, their clinical activity has generally been modest, with limited objective responses and frequent dose-limiting toxicities, including hyperglycemia, rash, and gastrointestinal effects, which have restricted their widespread clinical adoption [178]. Epigenetic therapies, such as HDAC inhibitors and DNA methyltransferase inhibitors, can restore expression of silenced tumor suppressor genes and sensitize tumors to chemotherapy or radiotherapy. Experimental approaches targeting microRNAs aim to inhibit oncogenic miRNAs, including miR-21 and miR-155, or to restore tumor-suppressive miRNAs, such as the miR-200 family [179]. Although these molecules are well characterized in OSCC biology, clinical translation remains limited, mainly due to challenges in delivery systems, molecular stability, and off-target effects, and therefore miRNA-based therapies remain largely in the experimental stage [180].

Molecular biomarkers and therapeutic targets in OSCC span genetic, epigenetic, protein, and non-coding RNA categories, offering applications in early detection, prognosis, and precision therapy. *TP53* mutation/ctDNA provides a minimally invasive approach for early diagnosis and monitoring of tumor burden [181]. Promoter hypermethylation of *CDKN2A* serves both as an early detection marker and a prognostic indicator [182]. Overexpression of EGFR, at the protein or genetic level, informs predictive responses to targeted therapies [141]. Dysregulated non-coding RNAs, including miR-21 in saliva or plasma and lncRNAs such as HOTAIR and MALAT1, act as diagnostic, prognostic, and metastasis-predictive biomarkers [183,184,185]. Key signaling pathways, notably PI3K/AKT/mTOR, represent actionable therapeutic targets, particularly in tumors with *PIK3CA* activation or *PTEN* loss [186]. Epigenetic therapies, including HDAC inhibitors, demonstrate the ability to reactivate silenced tumor suppressors and enhance responsiveness to conventional treatments [156].

While targeted therapies such as PI3K or EGFR inhibitors have faced challenges in clinical trials due to compensatory feedback loops, immune checkpoint inhibitors (ICIs) have revolutionized the therapeutic paradigm for advanced OSCC. Currently, the anti-PD-1 monoclonal antibodies Pembrolizumab and Nivolumab serve as the standard of care for recurrent or metastatic OSCC [187,188]. The clinical deployment of these agents relies heavily on the Combined Positive Score (CPS) to quantify PD-L1 expression within both tumor and infiltrating immune cells [188].

However, a critical evaluation of the multi-omics biomarkers discussed in this review (such as salivary miRNAs or ctDNA) reveals a significant gap between bench and bedside. The majority of these molecular candidates remain in preclinical or early-phase validation stages (Phase I/II) and are not yet integrated into international clinical guidelines (such as NCCN or ESMO) for routine screening or therapeutic monitoring. Translating these multi-omics signatures into robust, cost-effective, and standardized clinical assays remains a primary challenge for personalized OSCC management.

Together, these molecular insights underscore the potential of integrated biomarker-guided approaches for personalized management of OSCC. Key biomarkers and therapeutic targets in OSCC are summarized in Table 5.

### 9.3. Limitations of the Current Literature and Review

Although this review integrates multi-omics data to provide a holistic view of OSCC, several limitations must be acknowledged. First, as a narrative review, the literature selection is subject to potential search and selection biases compared to a formal systematic review. Second, a major confounding factor in current oncology literature is the frequent pooling of oral cavity data into broader HNSCC datasets, which restricts the precise isolation of oral-specific molecular profiles. Finally, high experimental heterogeneity regarding OSCC cell line authenticity, differing scoring criteria for biomarker expression (varying thresholds for miRNA upregulation), and distinct geographic etiologies (betel quid vs. tobacco) limit the immediate clinical translation of the summarized findings.

## 10. Conclusions

Oral squamous-cell carcinoma (OSCC) is a molecularly complex malignancy driven by the interplay of genetic, transcriptomic, epigenetic, and environmental factors. These mechanisms converge on interconnected signaling networks that promote tumor initiation, progression, metastasis, immune evasion, and therapeutic resistance, highlighting the biological heterogeneity of the disease.

Recent advances in multi-omics profiling have substantially improved our understanding of OSCC pathogenesis and have identified promising biomarkers and therapeutic targets. Although candidates such as circulating tumor DNA, DNA methylation signatures, and non-coding RNAs show considerable potential for early detection and personalized management, most remain investigational and require validation in large prospective clinical studies before routine implementation.

Future research should focus on integrating multi-omics data with clinical and pathological information to better define biologically relevant OSCC subtypes, clarify the role of the tumor microenvironment and oral microbiome, and overcome resistance to targeted therapies. Together with continued progress in early detection strategies, these advances are expected to support the development of more precise, individualized approaches that ultimately improve outcomes for patients with OSCC.

## Figures and Tables

**Table 1:** Recurrent Genetic Alterations in OSCC.

Gene	Type of Alteration	Approximate Frequency in OSCC (%)	Functional Impact	References
*TP53*	Somatic mutation	~50–70%	Loss of genomic integrity, apoptosis evasion	[68,77]
*CDKN2A*	Inactivation/deletion	~10–20%	Cell cycle deregulation	[46,69]
*HRAS*	Oncogenic point mutation	~5–20%	Constitutive proliferative signaling	[71,78]
*PIK3CA*	Activating mutation	~2–12%	PI3K/AKT pathway activation	[70,78]
*NOTCH1*	Variable dysregulation	~15–30%	Altered differentiation pathways	[72,78]
*FAT1*	Loss-of-function mutation	~15–35%	EMT promotion, invasion	[74,79]
*CASP8*	Inactivation	~8–23%	Impaired apoptosis	[73,79]
*EGFR*	Amplification/overexpression	30–90%	Enhanced proliferative and survival signaling	[75,80]
*MYC*	Amplification	~10–20%	Proliferation, metabolic reprogramming	[76,81]

**Table 2:** Representative Studies on Epigenetic Alterations in OSCC.

Epigenetic Mechanism	Genes/Molecules	Study/Sample	Key Findings	Reference
DNA methylation	*CDKN2A*, *RASSF1A*, *MLH1*	HNSCC/OSCC tumor tissues	Promoter hypermethylation silences tumor suppressors; correlates with tumor stage	[120]
Histone modification	HDAC1, HDACs, HMTs	OSCC cells and tissues	Dysregulated histone deacetylases repress pro-apoptotic genes; PRC2/EZH2-mediated H3K27me3 silences differentiation genes	[121]
miRNA dysregulation	miR-21, miR-155, miR-200 family	OSCC tissues vs. normal	miR-21 and miR-155 upregulated in OSCC; members of the miR-200 family downregulated, contributing to enhanced proliferation/invasion and EMT	[122]
lncRNA dysregulation	HOTAIR, MALAT1	CAL27	Overexpression of HOTAIR and MALAT1 promotes invasion, EMT and correlates with metastasis and poor prognosis	[123]

**Table 3:** Key Dysregulated Signaling Pathways in OSCC.

Pathway	Mechanism of Dysregulation	Functional Impact	References
EGFR-MAPK/PI3K/STAT	Overexpression/ligand activation	Proliferation, survival, immune evasion	[141]
PI3K/AKT/mTOR	PIK3CA mutations, *PTEN* loss	Apoptosis resistance, metabolism, angiogenesis	[142]
Wnt/β-catenin	β-catenin stabilization	EMT, stemness, invasion	[143]
JAK/STAT	Persistent STAT3 activation	Proliferation, anti-apoptosis, immune modulation	[144]

**Table 4:** Representative Genetic and Functional Studies in OSCC Cell Lines.

Study Focus	Cell Lines	Key Findings	References
Transcriptome profiling	SCC15, SCC25	Identification of somatic mutations and expression patterns, including *TP53* and CCND1 alterations	[152]
*NOTCH1* functional roles	CAL27, HSC-3	*NOTCH1* inhibition reduces proliferation and EMT markers	[153]
miRNA modulation	SCC25	miR-21 inhibition increases PTEN expression and is associated with reduced invasion and pro-tumor signaling	[154]
PIK3CA/AKT pathway inhibition	CAL27	PI3K inhibitors decrease proliferation and sensitize cells to apoptosis	[155]
Epigenetic therapy evaluation	OSCC cell lines (unspecified)	HDAC inhibitors (entinostat) restore tumor suppressor expression and reduce proliferation	[156]
Invasion and metastatic potential studies	HSC-2, HSC-4, Ca9-22	Frequently used highly invasive OSCC models for investigating migration, invasion, EMT, and metastasis-related mechanisms	[157,158]
Molecular and therapeutic response studies	HSC-2, HSC-4, Ca9-22	Commonly used Japanese-derived OSCC cell lines for evaluating signaling pathways, drug responses, and tumor biology	[159]

**Table 5:** Representative Biomarkers and Therapeutic Targets in OSCC.

Biomarker/Target	Type	Clinical Application	References
*TP53* mutation/ctDNA	Genetic	Early detection of recurrence and tumor monitoring via liquid biopsy	[181]
*CDKN2A* promoter methylation	Epigenetic	Early detection, prognosis	[182]
EGFR overexpression	Protein/Genetic	Predictive for targeted therapy	[141]
miR-21 (saliva/plasma)	miRNA	Diagnostic and prognostic biomarker	[185]
HOTAIR/MALAT1	lncRNA	Prognosis, metastasis prediction	[183,184]
PI3K/AKT/mTOR pathway	Signaling	Therapeutic target	[186]
HDAC inhibitors	Epigenetic therapy	Reactivate silenced tumor suppressors	[156]

## Data Availability

No new data were created or analyzed in this study. Data sharing is not applicable to this article.
